# RAUM-VO: Rotational Adjusted Unsupervised Monocular Visual Odometry

**DOI:** 10.3390/s22072651

**Published:** 2022-03-30

**Authors:** Claudio Cimarelli, Hriday Bavle, Jose Luis Sanchez-Lopez, Holger Voos

**Affiliations:** Interdisciplinary Center for Security Reliability and Trust (SnT), University of Luxembourg, 1855 Luxembourg, Luxembourg; hriday.bavle@uni.lu (H.B.); joseluis.sanchezlopez@uni.lu (J.L.S.-L.); holger.voos@uni.lu (H.V.)

**Keywords:** visual odometry, depth estimation, unsupervised learning, deep learning

## Abstract

Unsupervised learning for monocular camera motion and 3D scene understanding has gained popularity over traditional methods, which rely on epipolar geometry or non-linear optimization. Notably, deep learning can overcome many issues of monocular vision, such as perceptual aliasing, low-textured areas, scale drift, and degenerate motions. In addition, concerning supervised learning, we can fully leverage video stream data without the need for depth or motion labels. However, in this work, we note that rotational motion can limit the accuracy of the unsupervised pose networks more than the translational component. Therefore, we present RAUM-VO, an approach based on a model-free epipolar constraint for frame-to-frame motion estimation (F2F) to adjust the rotation during training and online inference. To this end, we match 2D keypoints between consecutive frames using pre-trained deep networks, Superpoint and Superglue, while training a network for depth and pose estimation using an unsupervised training protocol. Then, we adjust the predicted rotation with the motion estimated by F2F using the 2D matches and initializing the solver with the pose network prediction. Ultimately, RAUM-VO shows a considerable accuracy improvement compared to other unsupervised pose networks on the KITTI dataset, while reducing the complexity of other hybrid or traditional approaches and achieving comparable state-of-the-art results.

## 1. Introduction

One of the key elements for robot applications is autonomously navigating and planning a trajectory according to surrounding space obstacles. In the context of navigation systems, self-localization and mapping are pivotal components, and a wide range of sensors—from exteroceptive ones, such as the Global Positioning System (GPS), to proprioceptive ones, such as inertial measurement units (IMUs), as well as light detection and ranging (LiDAR) 3D scanners, and cameras—have been employed in the search for a solution to this task. As humans experience the rich amount of information coming from vision daily, exploring solutions that rely on a pure imaging system is particularly intriguing. Besides, relying only on visual clues is desirable as these are easy to interpret, and cameras are the most common sensor mounted on robots of every kind.

Visual simultaneous localization and mapping (V-SLAM) methods aim to optimize the tasks of motion estimation, that is, the 6 degrees of freedom (6DoF) transform that relates one camera frame to the subsequent one in 3D space, and 3D scene geometry (i.e., the depth and structure of the environment), in parallel. Notably, due to the interdependent nature of the two tasks, an improvement on the solution for one influences the other. On the one hand, the mapping objective is to maintain global consistency of the locations of the landmarks, that is, selected points of the 3D world that SLAM tracks. In turn, revisiting a previously mapped place may trigger a loop-closure [1], which activates a global optimization step for reducing the pose residual and smoothing all the past trajectory errors [2]. On the other hand, visual odometry (VO) [3] intends to carry out a progressive estimation of the ego-motion without the aspiration of obtaining a globally optimal path. As such, we can define VO as a sub-component of V-SLAM without the global map optimization routine required to minimize drift [4]. However, even VO methods construct small local maps composed by the tracked 2D features, to which a depth measurement is associated either through triangulation [5] or probabilistic belief propagation [6,7]. In turn, these 3D points are needed to estimate the motion between future frames.

Unsupervised methods have gained popularity for camera motion estimation and 3D geometry understanding in recent years [8]. Especially regarding monocular VO, approaches such as TwoStreamNet [9] have shown equally good or even superior performances compared to traditional methods, such as VISO2 [10] or ORB-SLAM [11]. The unsupervised training protocol [12] bears some similarities with the so-called direct methods [13]. Both approaches synthesize a time-adjacent frame by projecting pixel intensities using the current depth and pose estimations and minimizing a photometric loss function. However, the learned strategy differs from the traditional one because the network incrementally incorporates the knowledge of the 3D structure and the possible range of motions into its weights, giving better hypotheses during later training iterations. Moreover, through learning, we can overcome the typical issues of traditional monocular visual odometry. For example, the support of a large amount of example data during training can help solve degenerate motions (e.g., pure rotational motion), scale ambiguity and scale drift, initialization and model selection, low or homogeneously textured areas, and perceptual aliasing [4]. However, being aware of the solid theory behind the traditional methods [14] and their more general applicability, we leverage geometrical image alignment to improve the pose estimation.

**Figure 1:**
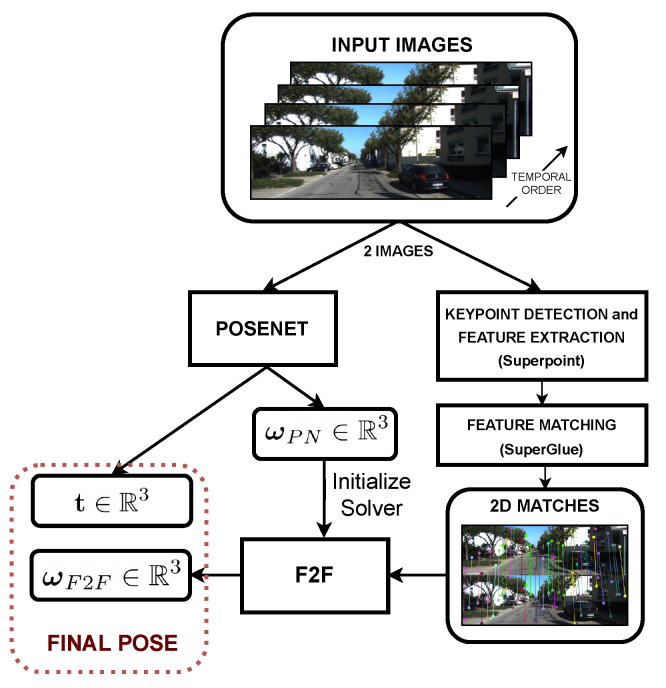
RAUM-VO block diagram. The figure shows the flow of information inside RAUM-VO from the input image sequence to the final estimated pose between each pair of consecutive image frames.

Therefore, in this work, we present RAUM-VO. Our approach, shown in Figure 1, combines unsupervised pose networks with two-view geometrical motion estimation based on a model-free epipolar constraint to correct the rotations. Unlike recent works [15,16] that train optical flow and use complex or computationally demanding strategies for selecting the best motion model, our approach is more general and efficient. First, we extract 2D keypoints using Superpoint [17] from each input frame and match the detected features from pairs of consecutive frames with Superglue [18]. Subsequently, we estimate the frame-to-frame motion using the solver proposed by Kneip et al. [19], which we name F2F, and use the rotation to guide the training with an additional self-supervised loss. Finally, RAUM-VO efficiently adjusts the rotation predictions with F2F during online inference, while retaining the scaled translation vectors from the pose network.

Our contributions are summarized as follows:

- We present RAUM-VO, an algorithm to improve the pose estimates of unsupervised pose networks for monocular odometry. To this end, we introduce an additional self-supervision loss using frame-to-frame rotation to guide the network’s training. Further, we adjust the rotation predicted by the pose network using the motion estimated by F2F during online inference to improve the final odometry.
- We compare our method with state-of-the-art approaches on the widely adopted KITTI benchmark. RAUM-VO improves the performance of pose networks and is comparably good as more complex hybrid methods, while being more straightforward to implement and more efficient.

## 2. Background on SLAM

The difference between SLAM and VO is the absence of a mapping module that performs relocalization and global optimization of the past poses. Aside from this aspect, we can consider contributions in monocular SLAM works seamlessly with those in the VO literature. A primary type of approach to SLAM is filter-based, either using extended Kalman filters (EKFs) (as in MonoSLAM [20]) or particle filters (as in FastSLAM [21]), and keyframe-based [5], referred in robotics to as smoothing [22]. This name entails the main difference between keyframe-based and filtering. While the first optimizes the poses and the landmarks associated with keyframes (a sparse subset of the complete history of frames) using batch non-linear least squares or bundle adjustment (BA) [23], the latter marginalizes past poses’ states to estimate the last at the cost of accumulating linearization errors [24]. In favor of bundle adjustment, Strasdat et al. [25] show that the accuracy of the pose increases when the SLAM system tracks more features and that the computational cost for filtering is cubic in the number of features’ observations, compared to linear for BA. Thus, using BA with an accurate selection of keyframes allows more efficient and robust implementations of SLAM. Unsupervised methods are more similar to the keyframe-based SLAM. The motion is not the result of a probabilistic model propagation and a single-step update but of an iterative optimization to align a batch of image measurements.

Motion estimation approaches fall into either direct or indirect categories based on the information or measurements included in the optimized error function. The direct method [13,26] includes intensity values in a non-linear energy function representing the photometric difference between pixels’ or patches’ correspondences. These are found by projecting points from one frame to another using the current motion and depth estimation, which is optimized either through the Gauss–Newton or Levenberg–Marquardt method. Instead, indirect methods [5,11] leverage epipolar geometry theory [14] to estimate motion from at least five matched 2D point correspondences, in the case of calibrated cameras [27], or eight, in the case of uncalibrated cameras [28]. After initializing a local map from triangulated points, perspective-n-point (PnP) [29] can be used with a random sample consensus (RANSAC) robust iterative fitting scheme [30] to obtain a more precise relative pose estimation. Subsequently, local BA refines the motion and the geometrical 3D structure by optimizing the reprojection error of the tracked features.

We do not apply the BA technique to correct the accumulated pose errors in this work. However, we investigate PnP motion estimation in place of the trained pose network and compare the results in Section 6.1.

## 3. Related Work

### Unsupervised Learning of Monocular VO

The pioneering work of Garg et al. [31] represents a fundamental advancement, because they approached the problem of depth prediction from a single frame in an unsupervised manner for the first time. Their procedure consists of synthesizing a camera’s depths in a rectified stereo pair by warping the other using the calibrated baseline and focal lengths. Godard et al. [32] use the stereo pair to enforce a consistency term between left and right synthesized disparities, while adopting the structural similarity (SSIM) metric [33] as a more informative visual similarity function than the $L_{1}$ loss. SfM-Learner [12] relies entirely on monocular video sequences and proposes the use of a bilinear differentiable sampler from ST-Nets [34] to generate the synthesized views.

Because the absolute metric scale is not directly observable from a single camera (without any prior knowledge about object dimensions), stereo image pairs are also helpful to recover a correct metric scale during training while maintaining the fundamental nature of a monocular method [35,36,37]. Mahjourian et al. [38] impose the scale consistency between adjacent frames as a requirement for the depth estimates by aligning the 3D point clouds using iterative closest point (ICP) and approximating the gradients of the predicted 6DoF transform. Instead, Bian et al. [39], arguing that the previous approach ignores second-order effects, show that it is possible to train a globally consistent scale with a simple constraint over consecutive depth maps, allowing one to reduce drift over long video sequences. In [40], a structure-from-motion (SfM) model is created before training and used to infer a global scale, using the image space distance between projected coordinates and optical flow displacements. More recently, several approaches [15,16,41] have leveraged learned optical flow dense pixel correspondences to recover up-to-scale two-view motion based on epipolar geometry. Therefore, they resolve the scale factor by aligning a sparse set of points with the estimated depths.

One of the main assumptions of the original unsupervised training formulation is that the world is static. Hence, many works investigate informing the learning process about moving objects through optical flow [42,43,44,45,46,47,48,49,50,51,52,53]. The optical flow, which represents dense maps of the pixel coordinates displacement, can be separated into two components. The first, the rigid flow, is caused by the camera’s motion. The second, the residual flow, is caused by dynamic objects that move freely in relation to the camera frame. Therefore, these methods train specific networks to explain the pixel shifts inconsistent with the two-view rigid motion. However, these methods focus principally on the depth and optical flow maps quality and give few details about the impact of detecting moving objects on the predicted two-view motion. Notably, they use a single metric to benchmark the relative pose that is barely informative about the global performance and cannot distinguish the improvements clearly.

A recent trend is to translate traditional and successful approaches such as SVO [54], LSD-SLAM [26], ORB-SLAM [11], and DSO [13] into their learned variants, or to take them as inspiration for creating hybrid approaches, where the neural networks usually serve as an initialization point for filtering or pose graph optimization (PGO) [55,56,57,58,59,60,61,62]. However, RAUM-VO focuses on improving the predicted two-view motion of the pose network without introducing excessive computation overhead as required by a PGO backend.

Instead of training expensive optical flow, RAUM-VO leverages a pre-trained Superpoint [17] network for keypoint detection and feature description and Superglue [18] for finding valid correspondences. Unlike optical flow, the learned features do not depend on the training dataset and generalize to a broader set of scenarios. In addition, using Superglue, we avoid heuristics for selecting good correspondences among the dense optical flow maps, which we claim could be a more robust strategy. However, we do not use any information about moving objects to discard keypoints lying inside these dynamic areas. Finally, differently from other hybrid approaches [15,16], we do not entirely discard the pose network output, but we look for a solution that improves its predictions efficiently and sensibly. Thus, the adoption of the model-free epipolar constraint of Kneip and Lynen [19] allows us to find the best rotation that explains the whole set of input matches without resorting to various motion models and RANSAC schemes. To the best of our knowledge, we are the first to test such an approach combined with unsupervised monocular visual odometry.

## 4. Method

**Figure 2:**
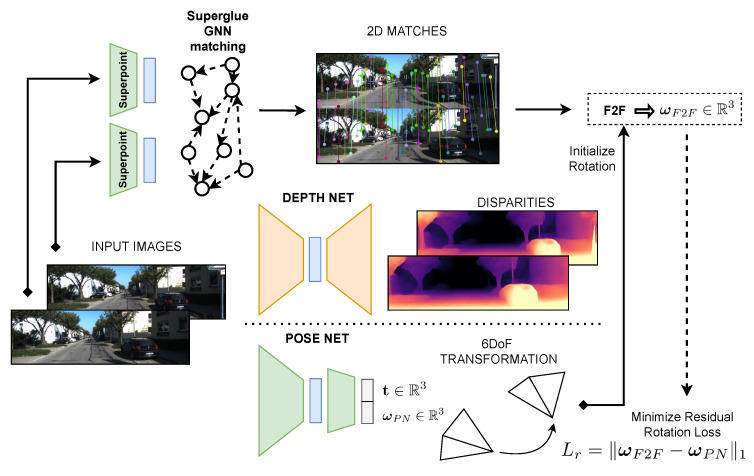
Diagram of RAUM-VO training. A sequence of images and 2D matches between pairs is the input for the training. The depth network takes only a single image to output a disparity map. The pose network outputs the 3D rigid transformation, as rotation and translation, between the two input images temporally ordered concatenated along the channel dimension. The matches are the input to the frame-to-frame rotation algorithm, whose output guides the training and adjusts the pose network estimation at test time.

This section outlines the proposed algorithm, RAUM-VO, for estimating the motion from a sequence of monocular camera images using a combination of deep neural networks and traditional epipolar geometry. This work follows Zhou et al. [12], who established an unsupervised training protocol based on view synthesis and photometric loss, which we describe in Section 4.1. In addition, to facilitate the learning process, we describe additional techniques implemented in our training in Section 4.2 and Section 4.3. As shown in Figure 2, the training outcome is a depth network that has learned to associate a disparity map to a single input image frame and a pose network that predicts the 6DoF rigid transformation between two consecutive frames. Additionally, we use the Superpoint [17] network to extract 2D keypoints descriptors. Consequently, using a pre-trained Superglue graph neural network (GNN) [18], RAUM-VO matches the corresponding features between pairs of successive frames. These matches are the input for the two-view motion estimation method [19] (see Section 4.4), whose rotation corrects the network’s output.

### 4.1. View Synthesis and Photometric Loss

The principle for obtaining a supervision signal shares some similarities with direct visual odometry [55]. Given two images at time *t* and t+1, $I_{t}$ and $I_{t+1}$, respectively, the depth network produces disparity (inverse depth) maps $d_{t}$ and $d_{t+1}$, respectively, and the pose network produces a 6DoF transformation. $T_{t\to t+1}=\left[\;R\;|\;t\;\right]$. Then, we obtain the depth maps $D_{t}$ and $D_{t+1}$ by inverting the disparities and normalizing them between a predefined minimum and maximum range limit. Finally, let K denote the intrinsic camera matrix, and $p_{t}=\left[u,v\right]$ a 2D pixel coordinate on $I_{t}$ image plane, in 2D homogeneous coordinates. The projection of $p_{t}$ into the reference frame of $I_{t+1}$, $p_{t\to t+1}$, is given by the following equation:

(1) $$p_{t\to t+1}=\pi \left(KT_{t\to t+1}K^{-1}H\left(p_{t},D_{t}\left[p_{t}\right]\right)\right)\;,$$

where $D_{t}\left[p_{t}\right]$ denotes the depth value at the point $p_{t}$, and H is the operation to lift the 2D pixel coordinates to 3D homogeneous coordinates:

(2) H:([u,v],z)↦[u∗z,v∗z,z,1]=[x,y,z,1],

while π is the projection to the image plane:

(3) π:([x,y,z,1])↦[x/z,y/z]=[u,v].

Using the (sub-)differentiable bilinear sampling operation, which we note with S, introduced with spatial transformer networks (STNs) [34], we obtain a synthesized version of $I_{t+1}$, $I_{t\to t+1}$, by interpolating its intensity values at the locations indicated by a grid of points $p_{t\to t+1}$.

(4) $$I_{t\to t+1}=S\left(I_{t+1},p_{t\to t+1}\right)\;.$$

Next, we optimize the estimated disparities and poses by minimizing the perceptual distance between the image $I_{t+1}$ and its synthesized version $I_{t\to t+1}$. Following the initial suggestion of [63] and the example of previous similar works [32,35], this distance is best assessed by a combination of L1 and SSIM [33], which is differentiable with respect to both depth and pose networks parameters. Particularly, the SSIM function aims to quantify the visual similarity of $I_{t+1}$ and its synthetic reconstruction $I_{t\to t+1}$ by comparing the luminance, contrast, and structure measurements on windows of size n×n.

Therefore, the *photometric loss* $L_{p}$, equates to:

(5) $$L_{p}=\;\alpha_{\mathrm{SSIM}}\frac{1-\mathrm{SSIM}(I_{t+1},I_{t\to t+1})}{2}\;+\;\alpha_{l_{1}}{∥I_{t+1}-I_{t\to t+1}∥}_{1}\;.$$

In our experiments, we set $\alpha_{\mathrm{SSIM}}=0.85$ and $\alpha_{l_{1}}=0.15$.

Notably, this warping mechanism succeeds with the assumptions that the scene is static, there are no occlusions, and the lighting conditions are constant, without reflections. Notwithstanding that the training process may be robust to minor violations of these assumptions, solutions for reducing dynamic objects [49] and non-Lambertian surfaces’ [62] impact on the optimization convergence have been provided in the recent literature. Instead, we rely on simpler mechanisms to alleviate the dynamic world conditions. During training, we extend the view synthesis procedure to the previous frame $I_{t-1}$ as well. Hence, we consider the minimum between $L_{p}\left(I_{t-1},I_{t}\right)$ and $L_{p}\left(I_{t-1},I_{t}\right)$ on a per-pixel basis as the final photometric loss. This strategy mitigates the effects of dis-occluded pixels [37].

To conclude, we would like to add a few observations. First, while the output would be random at the beginning, it is expected to converge to a meaningful value through the joint optimization process of the two networks. Next, the scale of the 6DoF transformation, foreseeably, reflects the depth scale, as they are jointly optimized. However, even if not aligned with the metric scale of the scene, it is plausibly globally consistent. Remarkably, this is an advantage over geometrical methods since, for the latter, we would need to take further precautions to avoid scale drifts [26,64]. In Section 4.3, we will introduce an additional loss term to reinforce a global consistency constraint during training.

### 4.2. Depth Smoothness Loss

The photometric loss is not informative with homogeneous or low-textured areas of an image, and the depth estimation problem becomes ill posed. The pixels in these regions can be associated with disparity values and still obtain a similar visual appearance for a fixed rigid transformation [37]. However, we can introduce a prior on the estimated depth maps that encourage smooth changes of the disparities inside these regions while discouraging the formation of holes. Thus, by considering the first (or second [55])-order gradients of the image as weighting terms, we allow sharp discontinuities to appear only in correspondence of edges [32].

Therefore, the following equation constitutes the *depth smoothness loss* $L_{s}$:

(6) $$L_{s}=\left|\partial_{x}d_{t}\right|e^{-\left|\partial_{x}I_{t}\right|}+\left|\partial_{y}d_{t}\right|e^{-\left|\partial_{y}I_{t}\right|}\;,$$

where $\partial_{x}$ and $\partial_{y}$ are the first derivatives of the color image and disparity map taken along *x* and *y* directions.

### 4.3. Depth Consistency Loss

An issue of monocular VO, famously, is the non-observability of the metric scale of the surrounding environment and, consequently, of the motion between two views. This limitation leads to the well-known issue of scale drift, which has been successfully addressed in traditional BA-SLAM by performing the pose graph optimization over 3D similarity transforms [26,64]. From the perspective of learned mono-VO, Tateno et al. [65] explore the path of predicting depth maps using CNNs, confident of their capability to reproduce the metric scale passed through the ground-truth depths supervision. On the other hand, without depth supervision, an alternative approach to learning a metrically scale-aware network is from information regarding the translation vectors norm, as in [66], where the authors impose a velocity loss. Even though we cannot obtain the real scale during training, ensuring the depth consistency is fundamental for reducing the drift and easing the task of aligning the estimated trajectory with an external metric map. Therefore, in this work, lacking the knowledge of real-world scale and ground-truth depths, we adopt the loss for imposing depth consistency between two frames introduced by Bian et al. [39]. The following equation defines the *depth consistency loss* $L_{dc}$:

(7) $$L_{dc}=\frac{\left|D_{a\to b}-D_{b}\right|}{D_{a\to b}+D_{b}}\;,$$

where $D_{a\to b}$ represent the synthesized version of the depth estimated for image $I_{a}$ to the camera reference of image $I_{b}$ by means of the estimated pose $T_{a\to b}$ and the bilinear sampler.

### 4.4. F2F: Frame-to-Frame Motion

Here, we describe the pivotal component of our proposed method. In particular, we incorporate the rotation optimization formulated by Kneip and Lynen [19]. They propose an alternative epipolar constraint that enables one to solve the relative pose problem without many of the issues encountered in essential-matrix-based methods. Namely, these are:

- the indirect parametrization of the motion that has to be decomposed from the essential matrix, as in [14]:
  (8) $$E={\left[t\right]}_{x}\;R\;;$$
- multiple solutions from the decomposition that have to be disambiguated through a cheirality check and hence by triangulation;
- degenerate solutions that may result from either points lying on a single planar surface, distribution of the points in a small image area, and pure translational or rotational motion. In these cases, one approach is to select a different motion model, e.g., the homography matrix, after identifying the degeneracy with a proper strategy.

Therefore, given a set of image points $\left(p_{i},p_{i}^{\prime }\right)$ matched between two views, we translated them into pairs of unit-bearing vectors $\left(f_{i},f_{i}^{\prime }\right)$ through normalization. These vectors ideally start from the camera center and point in the direction of the corresponding 3D points, and each pair defines an epipolar plane. Then, the authors observe that the all the normal vectors of the epipolar planes need to be coplanar [67]. The normal vectors form together a 3-by-*n* matrix $N=[n_{1}\;\ldots \;n_{n}]$, and are defined as follows:

(9) $$n_{i}=f_{i}\times Rf_{i}^{\prime }\;.$$

Due to the coplanarity constraint, the covariance matrix ${\mathrm{NN}}^{T}=\;M$ has to be at most of rank 2. Notably, the problem is equivalent to a rank minimization parametrized by R, and is solved by finding the matrix M with the smallest minimum eigenvalue:

(10) $$R\;=\;\underset{R}{\arg\min}\lambda_{M,\min}\;.$$

Furthermore, the authors observe that the eigenvector associated with $\lambda_{M,\min}$ corresponds to the translation direction vector. Therefore, this method, which we name **F2F**, is able to retrieve the full frame-to-frame motion.

The problem is solved with a Levenberg–Marquardt procedure. To avoid the possible presence of local minima typical of non-linear optimization, we use the rotation estimated by the pose network as a starting point. In Section 6.1, we show the benefits of this initialization. In addition, we choose to perform a single optimization with all the matches instead of multiple RANSAC iterations. For restricting the number of matches outliers, we set the threshold of the Superglue match confidence score to 0.9. At the moment, we found that this approach works best for the data at hand after empirical evaluation of multiple RANSAC settings and inlier criteria.

Lastly, we include the rotation $R_{F2F}$ as supervision for the rotation output of the pose network, $R_{\mathrm{PN}}$, in the *residual rotation loss*$L_{r}$. To this aim, we map the rotation matrices into their axis-angle counterparts through the logarithm function:

(11) log:SO(3)→so(3);R↦log(R),

where so(3) is the Lie algebra associated to the Lie group of 3D rotations SO(3) [68]. Based on the isomorphism between so(3) and $R^{3}$ with the cross product, we treat the logarithm of a rotation matrix as a vector $\omega \in R^{3}$ decomposed into a unit-norm direction vector $u\in R^{3}$, representing the rotation axis, and its $L_{2}$ norm θ∈R, where θ∈[0,π] represents the angle of rotation:

(12) log(R)=ω=θu.

Therefore, we can compute the $L_{1}$ norm, denoted by ${∥\cdot ∥}_{1}$, of the distance between the rotation vector predicted by the network, $\omega_{PN}$, and the one estimated by F2F, $\omega_{F2F}$. Thus, we obtain the following *residual rotation loss*$L_{r}$:

(13) $$L_{r}={∥\omega_{F2F}-\omega_{PN}∥}_{1}\;.$$

In Figure 2, we show how all the components we described interact during the training of RAUM-VO.

The implementation of F2F used in this work is the one provided by the OpenGV library [69].

## 5. Experiments

This section provides details regarding our experimental procedure and the settings for accurately reproducing our results. In addition, we provide the results of VO obtained on KITTI and compare them with state-of-the-art methods.

### 5.1. Training Procedure

Because we have experienced a degradation in performance when including the $l_{dc}$ term early in training, we split it into two phases. Particularly, when the depth network has not yet found a convergence direction for a plausible geometrical structure, the $l_{dc}$ term, especially if it has a magnitude outweighing the photometric loss norm, could cause the depth maps to collapse towards a local minimum during the initial training phase. An alternative solution may be to adaptively adjust the weighing of $l_{dc}$ based on the value of $l_{p}$. Therefore, we add the depth consistency loss after the convergence of the photometric loss. In addition, we add the contribution of the loss $l_{r}$ in the second training phase to let the pose network reach an initial convergence plateau first.

Consequently, we obtain two models:

- **Simple-Mono-VO** is obtained after the first training phase by selecting the checkpoint with the best $t_{err}$ on the training set;
- **RAUM-VO** is obtained after the second phase by selecting the checkpoint with the best $t_{err}$ on the training set and correcting the rotations with the output of F2F.

### 5.2. Networks Architectures

The depth network has an encoder-decoder architecture [70] with skip connection similar to DispNet [71] used by SfM-Learner [12]. Specifically, the encoder is a ResNet18 [72], and the decoder has five layers of 3 × 3 convolutions followed by an ELU activation function [73], an up-sampling, and a concatenation with the “connected” encoder feature. In accordance with [39], we avoid multi-scale training for efficiency purposes. Therefore, we apply the sigmoid function to the last output to obtain a disparity map.

The pose network consists of one ResNet18 [72] encoder that takes as input a pair of images concatenated along the channel dimension. The feature extracted by the last layer is then the input to a small CNN decoder composed by:

1. one linear layer that reduces the feature to a 256-dimensional vector followed by ReLU [74] non-linearity;
2. two convolutional layers with 256 kernels of size 3 × 3 followed by ReLu non-linearities;
3. one linear layer that outputs the 6DoF pose vector as the vector $x\in R^{6}$, which contains the concatenation of the translation $t\in R^{3}$ and the axis-angle rotation $\omega_{PN}\in R^{3}$.

The network architectures are based on the Monodepth2 implementation [37] and use PyTorch [75]. Both networks encoders are initialized with pre-trained weights on the ImageNet dataset [76].

### 5.3. Experimental Settings

The images are resized to 640×192 before entering the network. During training, we sample with repetition 2000 images for each epoch. We use standard color image augmentation by slightly changing saturation, brightness, contrast, and hue, as in [37], and horizontal flipping. For the optimization, we use Adam [77] with parameters $\beta_{1}=0.9$ and $\beta_{2}=0.999$, and a learning rate $lr=10^{-4}$. We halve the learning rate when the loss does not decrease for 10 epochs. We keep the training until convergence of the loss or for at most 1800 epochs. The *depth smoothness loss*, *depth consistency loss*, and *residual rotation loss* weighing factors are 10^−3^, $5\times 10^{-1}$, and 1, respectively.

### 5.4. KITTI Results

We evaluate our visual odometry network on the KITTI odometry dataset [78]. To this aim, we use the sequences from 0 to 8 for training and the sequences 9 and 10 for testing. Furthermore, we use the tool provided by the author of DF-VO [16] to make sure we apply the same criteria for evaluation. Notably, we evaluate with the “7DoF alignment” setting that computes the similarity transform that best aligns the predicted trajectory with the ground truth using the Umeyama algorithm [79].

**Figure 3:**
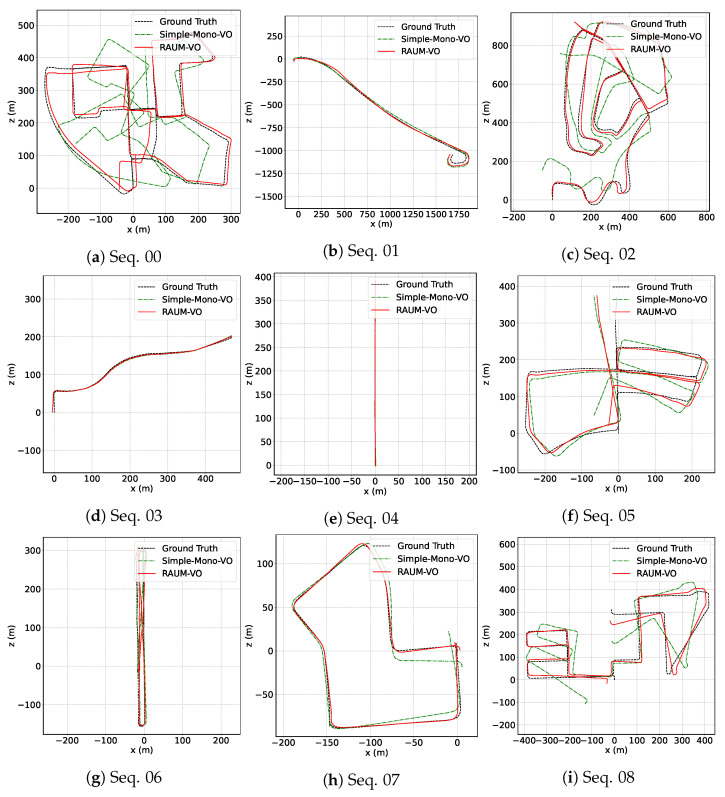
KITTI train trajectories. Estimated trajectories for the KITTI odometry sequences from 00 to 08. Poses are given in camera frame. Thus, positive *x* means right direction and positive *z* means forward. Best viewed in color.

**Figure 4:**
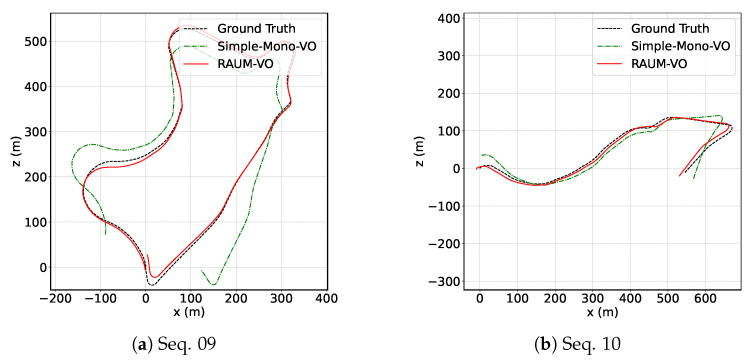
KITTI test trajectories. Estimated trajectories for the KITTI odometry sequences 09 and 10. Poses are given in camera frame. Thus, positive *x* means right direction and positive *z* means forward. Best viewed in color.

In Figure 3, we show the plots of the trajectories for the training sequences predicted by our two models and the ground-truth poses. By comparing these with the testing sequences displayed in Figure 4, we can appreciate the generalization capability of the neural network to unseen sequences, even if KITTI contains images from similar scenarios. Then, in Table 1, we compare our results with two pure geometrical approaches, ORB-SLAM [11] and VISO2 [10]; two unsupervised networks methods, SfM-Learner [12] and SC-SfMLearner [39]; and with the hybrid approach DF-VO [16]. For the evaluation, we use data from [16]. We note that the reported results for [39] are slightly different from the one in the paper and may refer to training with additional data. For our evaluation, we select those works that use only monocular image sequence during training and evaluation phases, as RAUM-VO does, because stereo image pairs give an unfair advantage to the depth reconstruction and, consequently, to the pose estimation, as documented in the literature [37]. Another condition for the evaluation regards the architectures of the depth and pose networks. Therefore, we selected methods in the learned categories that use comparable, if not equal, deep networks. Unfortunately, this is one element of discrepancy among the works in the literature of unsupervised pose and depth estimation, and that has to be taken into account when making comparisons.

While RAUM-VO does not surpass DF-VO performances in many sequences, its accuracy is comparable while being more efficient. Because DF-VO is one of the most promising hybrid approaches using monocular images for the VO, in Section 6.2, we examine the differences and advantages of our method in more detail. Regarding traditional methods, the average error of RAUM-VO is generally lower, except for the $r_{err}$ metric computed on ORB-SLAM only. However, unlike ORB-SLAM, we do not apply local BA. Regarding the unsupervised pose networks category, the proposed RAUM-VO proves to reduce the error effectively with the proposed rotation adjustment step. In the link (https://youtu.be/4woTiJRCrUI, accessed on 10 February 2022), we provide a video that shows the depth map predictions for all the KITTI sequences.

## 6. Discussion

Herein, we discuss and analyze the characteristics of RAUM-VO. First, in Section 6.1, we consider the rotational and translational components of the pose error separately to argue that the rotations offer a larger space to decrease the absolute trajectory error (ATE) shown in Table 1. In turn, this motivates the adoption of a specific measure to adjust the predicted rotations. Hence, we demonstrate how the pose network plays a valuable role in initializing the F2F solver. Lastly, in Section 6.2, we speculate on the factor that contributes the most to the accuracy of DF-VO compared to our approach.

### 6.1. General Considerations

In Table 2, we show that by modifying the simple-mono-VO predictions using the ground truth of either the translation or the rotation, there is a larger margin for improvement enclosed in the current rotation estimates than in the translational component of the error. We presume that this behavior is because we optimize translations directly on their vector space, contrary to the rotations. The manifold of rotations, *special orthogonal* group SO(3), only locally resembles a Euclidean topology [80] and needs intermediate representations to enable the optimization with gradient descent methods. As such, the axis-angles are a many-to-one mapping with SO(3), and alternative representations may be easier to approximate with a neural network [81]. In addition, the linear distance metric between translation vectors is easier to approximate than the non-linear counterparts for the SO(3) group [82]. Nevertheless, the rotation provided by the pose network is a better initialization point for the F2F than the identity or constant motion assumption. The results of the different types of initialization are visible in Table 3. By this, the pose network’s predicted rotations are always the best option for initializing the F2F solver and are paired only by constant motion assumption in some cases.

Then, we suggest that the pose network can regress the motion even in difficult motion situations, assuming that the depth network has learned a valid geometric structure. The pose and depth outcomes are strongly entangled due to their joint training, even if produced by separate networks. However, more precisely, we note that the performance of one component may be restricted by the other. While this may seem a trivial conclusion, it is necessary to clarify the limitations of this approach and bring us to the last reflection. We evaluate the odometry poses obtained by PnP combined with the depth network to prove our argument. To this aim, we back-project to 3D coordinates the matches in one view frame, the same utilized for our RAUM-VO, by interpolating the depth map values with the bilinear sampler of STNs.

Consequently, we can apply PnP with RANSAC to estimate the two view motions for all the sequences. Remarkably, the outcome of PnP, on average, matches closely that of the pose network (see Table 4), especially for the training sequences when we fix the rotation with F2F. This result aligns with those of, for example, DeepMatchVO [83] or DF-VO [16], which do not obtain significantly better odometry results by leveraging PnP directly during the training or at the test time. Interestingly, though, the combination of a PnP with the estimated depths works best for the test sequences, indicating that this approach may generalize more.

### 6.2. Comparison with DF-VO

We can probably ascribe the success of DF-VO to an accurately trained optical flow, which provides a significantly higher number of precise matches, in the order of thousands. Still, these correspondences are specific to the scenario they use to train the optical flow network. Conversely, the 2D features detected by Superpoint are fast to compute, distinctly identified, repeatable, and, more importantly, sparser (a few hundred). Therefore, we note that the optical flow network can hardly reach the generalization capability of a dedicated feature extraction network. Additionally, due to dense but noisy correspondences, DF-VO needs to iteratively search the best fit mode (e.g., based on the number of inliers), and decide between the essential or homography motion model with multiple RANSAC routines. While this approach accurately describes the two-view motion of the KITTI sequences, it turns out to be computationally expensive. Instead, RAUM-VO uses all the matches found by Superglue for solving the eigenvalue minimization problem of F2F only once, adding minimal overhead to the pose network run-time. Thus, we remove the need for repeated samples of the correspondences and avoid the numerous estimation of homography and essential matrices with the related model selection strategy. Therefore, we resort to the output of the pose network and a single model-free rotation adjustment step, which is comparably a more efficient approach.

Furthermore, another potential determining factor of success is the depth scale consistency. DF-VO considers the depth maps as a source of multiple hypotheses for the translation vector scale. Thus, we can presume that the disparities jointly learned with the optical flow have a higher degree of long-term scale consistency and structure accuracy. In this way, the DF-VO scale alignment procedure can recover the best norm for the translation vector, which the employed Nister 5-point [27] algorithm delivers only up to a scale factor. In addition, the depth consistency loss may not be as effective as the consistency loss between rigid motion and optical flow in maintaining a unique long-term scale factor.

Consequently, for evaluating our depth scale consistency, we applied a scale alignment procedure similar to DF-VO for scaling the translation solutions obtained from the F2F and essential matrix, using the implementations of OpenGV [69] and OpenCV, respectively. Notably, we pick the essential matrix with the most inliers after ten iterations, sampling each time 20% of the matches and estimating it using RANSAC with threshold $10^{-3}$. Next, we triangulate the 2D correspondences and keep only those that pass the cheirality check. Finally, we sample 80% of the triangulated points $X_{t}$ ten times and fit a linear model with RANSAC:

(14) $$Y_{d}=s\;X_{t}$$

to find the coefficient *s* that maps $X_{t}$ to $Y_{d}$, which is the set of 3D points obtained by projecting the matches with the estimated depths. Finally, we take the scale *s* that has the minimum $\delta ={∥1-s∥}_{2}$. We fall back to the pose-network-estimated translation only if less than 51% of matches do not pass the cheirality check or if $\delta >5\times 10^{-1}$. We accept the F2F or essential matrix translation in 93–97% of the cases with these loose constraints. We present the result of this test in Table 5. Still, we could not obtain a better translation than the pose network’s output. Besides, the multiple RANSAC routines and sampling matches from dense correspondences may grant a decisive advantage to DF-VO. We leave a deeper analysis to understand the factors at stake for future works.

## 7. Conclusions

In this paper, we have presented our approach, RAUM-VO, that combines the translation predicted by a pose network with the rotations estimated by a geometrical method named F2F. In practice, we introduced an additional self-supervised loss to guide the training. More importantly, during online inference, we adjust the rotations predicted by the pose network with a single estimation of F2F, avoiding complex strategies for model selection and multiple RANSAC loops. In addition, RAUM-VO uses Superpoint with Superglue to find robust 2D correspondences in place of randomly sampling optical flow, thus reducing training time and generalizing to more environments. Finally, we evaluated RAUM-VO on the KITTI odometry dataset and compared it with other relevant state-of-the-art methods. While efficient, this adjustment step is decisive for improving the prediction of unsupervised pose networks.

Future works can track or match the Superpoint features, using the associated descriptors, over longer frame distances, enabling local or global BA with loop closures similar to ORB-SLAM. More interestingly, the extension of F2F to multiple views, proposed by Lee and Civera [84], could be an alternative to rotation averaging [85] to initialize the pose graph optimization [86] together with the pose network prediction.

## Figures and Tables

**Table 1:** Odometry quantitative evaluation. Result obtained on KITTI odometry seq. 00–10. Data is retrieved from [16]. Best results are highlighted in **bold**, second best with an underline.

Category	Method	Metric	00	01	02	03	04	05	06	07	08	09	10	Train Avg. Err.	Tot. Avg. Err.
Geometric	ORB-SLAM2 [11] (w/o LC)	$t_{err}$	11.43	107.57	10.34	**0.97**	**1.30**	9.04	14.56	9.77	11.46	9.30	2.57	19.604	17.119
		$r_{err}$	**0.58**	0.89	**0.26**	**0.19**	**0.27**	0.26	**0.26**	0.36	**0.28**	0.26	**0.32**	**0.372**	**0.357**
		ATE	40.65	502.20	47.82	**0.94**	**1.30**	29.95	40.82	16.04	43.09	38.77	5.42	80.312	69.727
		RPE (m)	0.169	2.970	0.172	0.031	0.078	0.140	0.237	0.105	0.192	0.128	0.045	0.455	0.388
		RPE (°)	0.079	0.098	0.072	0.055	0.079	0.058	0.055	0.047	0.061	0.061	0.065	0.067	0.066
	VISO2 [10]	$t_{err}$	10.53	61.36	18.71	30.21	34.05	13.16	17.69	10.80	13.85	18.06	26.10	23.373	23.138
		$r_{err}$	2.73	7.68	1.19	2.21	1.78	3.65	1.93	4.67	2.52	1.25	3.26	3.151	2.988
		ATE	79.24	494.60	70.13	52.36	38.33	66.75	40.72	18.32	61.49	52.62	57.25	102.438	93.801
		RPE (m)	0.221	1.413	0.318	0.226	0.496	0.213	0.343	0.191	0.234	0.284	0.442	0.406	0.398
		RPE (°)	0.141	0.432	0.108	0.157	0.103	0.131	0.118	0.176	0.128	0.125	0.154	0.166	0.161
Unsupervised	SfM-Learner [12]	$t_{err}$	21.32	22.41	24.10	12.56	4.32	12.99	15.55	12.61	10.66	11.32	15.25	15.169	14.826
		$r_{err}$	6.19	2.79	4.18	4.52	3.28	4.66	5.58	6.31	3.75	4.07	4.06	4.584	4.490
		ATE	104.87	109.61	185.43	8.42	3.10	60.89	52.19	20.12	30.97	26.93	24.09	63.956	56.965
		RPE (m)	0.282	0.660	0.365	0.077	0.125	0.158	0.151	0.081	0.122	0.103	0.118	0.225	0.204
		RPE (°)	0.227	0.133	0.172	0.158	0.108	0.153	0.119	0.181	0.152	0.159	0.171	0.156	0.158
	SC-SfMLearner [39]	$t_{err}$	11.01	27.09	6.74	9.22	4.22	6.70	5.36	8.29	8.11	7.64	10.74	9.638	9.556
		$r_{err}$	3.39	1.31	1.96	4.93	2.01	2.38	1.65	4.53	2.61	2.19	4.58	2.752	2.867
		ATE	93.04	85.90	70.37	10.21	2.97	40.56	12.56	21.01	56.15	15.02	20.19	43.641	38.907
		RPE (m)	0.139	0.888	0.092	0.059	0.073	0.070	0.069	0.075	0.085	0.095	0.105	0.172	0.159
		RPE (°)	0.129	0.075	0.087	0.068	0.055	0.069	0.066	0.074	0.074	0.102	0.107	0.077	0.082
	**Simple-Mono-VO** **(Ours)**	$t_{err}$	9.365	8.920	6.830	3.697	2.570	4.964	3.138	3.568	7.125	13.625	11.131	5.575	6.812
		$r_{err}$	2.840	0.562	1.582	2.478	0.566	2.083	0.959	1.866	2.608	3.146	4.784	1.727	2.134
		ATE	94.949	30.004	83.155	4.112	2.377	30.227	8.726	8.872	59.887	66.591	18.792	35.812	37.063
		RPE (m)	0.090	0.304	0.087	0.037	0.055	0.041	0.051	0.044	0.074	0.166	0.077	0.087	0.093
		RPE (°)	0.072	**0.042**	0.057	0.048	0.036	0.049	0.040	0.048	0.052	0.067	0.083	0.049	0.054
Hybrid	DF-VO [16] (Mono)	$t_{err}$	**2.33**	39.46	3.24	2.21	1.43	**1.09**	**1.15**	**0.63**	**2.18**	**2.40**	**1.82**	5.969	5.267
		$r_{err}$	0.63	**0.50**	0.49	0.38	0.30	**0.25**	0.39	**0.29**	0.32	**0.24**	0.38	0.394	0.379
		ATE	**14.45**	117.40	19.69	1.00	1.39	**3.61**	**3.20**	**0.98**	**7.63**	**8.36**	**3.13**	18.817	16.440
		RPE (m)	**0.039**	1.554	0.057	**0.029**	**0.046**	**0.024**	**0.030**	**0.021**	**0.041**	**0.051**	**0.043**	0.205	0.176
		RPE (°)	**0.056**	0.049	**0.045**	**0.038**	**0.029**	**0.035**	**0.029**	**0.030**	**0.037**	**0.036**	**0.043**	**0.039**	**0.039**
	**RAUM-VO** **(Ours)**	$t_{err}$	2.548	**8.354**	**2.578**	3.217	2.860	3.045	3.033	2.390	3.632	2.927	5.843	**3.517**	**3.675**
		$r_{err}$	0.775	0.868	0.582	1.334	0.645	1.153	0.837	1.037	1.074	0.318	0.683	0.923	0.846
		ATE	16.272	**23.748**	**16.139**	2.602	2.283	17.470	9.234	2.164	16.303	8.664	12.297	**11.802**	**11.561**
		RPE (m)	0.040	**0.257**	**0.050**	0.030	0.052	0.038	0.046	0.028	0.053	0.068	0.078	**0.066**	**0.067**
		RPE (°)	0.059	0.062	0.048	0.048	0.035	0.044	0.042	0.058	0.045	0.042	0.051	0.049	0.049

**Table 2:** The table shows an insight into the possible margins for improvement in the pose predictions coming from unsupervised methods. Hence, we substitute alternately the ground-truth translations and rotations in the pose network estimates. We show the variation in the relevant metrics for the KITTI test sequences 9 and 10.

	Metrics	09	10
Simple-Mono-VO	$t_{err}$	13.625	11.131
	$r_{err}$	3.146	4.784
	ATE	66.591	18.792
	RPE (m)	0.166	0.077
	RPE (°)	0.067	0.083
Ground-Truth Translation	$t_{err}$	13.325	11.409
	$r_{err}$	3.146	4.784
	ATE	65.081	20.715
	RPE (m)	0.162	0.028
	RPE (°)	0.067	0.083
Ground-Truth Rotation	$t_{err}$	3.029	6.038
	$r_{err}$	0.010	0.014
	ATE	9.026	12.894
	RPE (m)	0.070	0.080
	RPE (°)	0.005	0.005

**Table 3:** F2F solver initialization. Comparison of different initialization approaches for the Levenberg–Marquardt scheme that solves the frame-to-frame motion. Overall, the rotation from the pose network is the best, followed by a constant motion model.

Initialization	Metrics	00	01	02	03	04	05	06	07	08	09	10	Avg. Train	Avg. All
Identity	$t_{err}$	6.192	8.023	5.888	3.919	2.860	7.659	9.100	10.969	5.402	3.851	9.475	6.668	6.667
	$r_{err}$	2.222	1.025	1.670	1.909	0.645	3.340	2.926	6.565	1.926	0.742	2.605	2.470	2.325
	ATE	39.195	21.231	91.621	2.651	2.283	40.192	19.682	20.592	30.142	12.939	13.399	29.732	26.721
	RPE (m)	0.040	0.259	0.060	0.030	0.052	0.039	0.046	0.036	0.052	0.069	0.077	0.068	0.069
	RPE (°)	0.100	0.101	0.072	0.082	0.035	0.083	0.059	0.158	0.067	0.070	0.088	0.084	0.083
Constant Motion	$t_{err}$	6.062	12.009	5.823	6.606	2.860	5.877	3.033	2.481	19.533	3.255	5.843	7.143	6.671
	$r_{err}$	2.128	1.833	1.728	3.119	0.645	2.105	0.837	1.150	7.772	0.862	0.683	2.368	2.078
	ATE	58.308	49.099	79.710	6.678	2.283	29.920	9.234	2.258	99.024	11.190	12.297	37.390	32.727
	RPE (m)	0.044	0.265	0.056	0.030	0.052	0.039	0.046	0.028	0.160	0.069	0.078	0.080	0.079
	RPE (°)	0.075	0.086	0.059	0.066	0.035	0.060	0.042	0.068	0.702	0.072	0.051	0.133	0.120
Pose Network (RAUM-VO)	$t_{err}$	2.548	8.354	2.578	3.217	2.860	3.045	3.033	2.390	3.632	2.927	5.843	3.517	3.675
	$r_{err}$	0.775	0.868	0.582	1.334	0.645	1.153	0.837	1.037	1.074	0.318	0.683	0.923	0.846
	ATE	16.272	23.748	16.139	2.602	2.283	17.470	9.234	2.164	16.303	8.664	12.297	11.802	11.561
	RPE (m)	0.040	0.257	0.050	0.030	0.052	0.038	0.046	0.028	0.053	0.068	0.078	0.066	0.067
	RPE (°)	0.059	0.062	0.048	0.048	0.035	0.044	0.042	0.058	0.045	0.042	0.051	0.049	0.049

**Table 4:** PnP vs. pose network. Comparison of the trajectory estimated by PnP combined with the depth network and the poses predicted by our trained network.

Poses Source	Metrics	00	01	02	03	04	05	06	07	08	09	10	Avg. Train	Avg. All
Pose Network (Simple-Mono-VO)	$t_{err}$	9.365	8.920	6.830	3.697	2.570	4.964	3.138	3.568	7.125	13.625	11.131	5.575	6.812
	$r_{err}$	2.840	0.562	1.582	2.478	0.566	2.083	0.959	1.866	2.608	3.146	4.784	1.727	2.134
	ATE	94.949	30.004	83.155	4.112	2.377	30.227	8.726	8.872	59.887	66.591	18.792	35.812	37.063
	RPE (m)	0.090	0.304	0.087	0.037	0.055	0.041	0.051	0.044	0.074	0.166	0.077	0.087	0.093
	RPE (°)	0.072	0.042	0.057	0.048	0.036	0.049	0.040	0.048	0.052	0.067	0.083	0.049	0.054
PnP	$t_{err}$	6.808	17.627	6.319	4.046	2.627	4.629	2.981	3.013	6.360	7.019	6.708	6.045	6.194
	$r_{err}$	2.190	1.195	1.339	2.364	0.582	1.863	0.781	1.691	2.317	2.029	2.644	1.591	1.727
	ATE	79.125	63.596	76.800	4.402	2.424	29.000	8.660	7.106	52.700	35.664	9.576	35.979	33.550
	RPE (m)	0.061	0.636	0.086	0.033	0.055	0.039	0.049	0.040	0.067	0.082	0.073	0.118	0.111
	RPE (°)	0.060	0.057	0.049	0.042	0.029	0.039	0.032	0.036	0.043	0.068	0.085	0.043	0.049
F2F rotation w/ PnP translation	$t_{err}$	2.796	15.552	2.775	3.482	3.123	3.008	3.164	2.373	3.876	3.072	4.343	4.461	4.324
	$r_{err}$	0.775	0.868	0.582	1.334	0.645	1.146	0.837	0.861	1.074	0.318	0.683	0.902	0.829
	ATE	17.662	41.782	15.194	2.342	2.459	17.203	9.451	3.983	16.741	8.288	8.909	14.091	13.092
	RPE (m)	0.043	0.527	0.053	0.034	0.055	0.040	0.050	0.035	0.055	0.071	0.073	0.099	0.094
	RPE (°)	0.059	0.062	0.048	0.048	0.035	0.045	0.042	0.059	0.046	0.042	0.051	0.049	0.049
F2F rotation w/ Pose Network translation (RAUM-VO w/o $L_{r}$)	$t_{err}$	2.829	9.870	2.766	4.146	3.080	3.029	3.177	2.802	3.804	3.130	5.875	3.945	4.046
	$r_{err}$	0.775	0.868	0.582	1.334	0.645	1.146	0.837	0.861	1.074	0.318	0.683	0.902	0.829
	ATE	18.339	28.499	15.497	2.468	2.419	17.363	9.502	4.732	16.426	9.033	12.410	12.805	12.426
	RPE (m)	0.043	0.307	0.053	0.037	0.055	0.041	0.051	0.036	0.056	0.070	0.079	0.075	0.075
	RPE (°)	0.059	0.062	0.048	0.048	0.035	0.045	0.042	0.059	0.046	0.042	0.051	0.049	0.049

**Table 5:** Scale alignment. Results of the scale alignment procedure applied to the translation vector from the F2F and the essential matrix estimated motions.

	Metrics	09	10
F2F Translation	$t_{err}$	4.14	5.68
	ATE	12.91	11.67
	RPE (m)	0.114	0.091
Essential Matrix Translation	$t_{err}$	4.02	5.99
	ATE	11.77	12.42
	RPE (m)	0.124	0.099
Pose Network (RAUM-VO)	$t_{err}$	2.927	5.843
	ATE	8.664	12.297
	RPE (m)	0.068	0.078

## Data Availability

In this work, we use the KITTI Odometry dataset that is publicly available at http://www.cvlibs.net/datasets/kitti/eval_odometry.php, accessed on 14 March 2022.
